# Long-Term Experience with Anaphylaxis and Desensitization to Alglucosidase Alfa in Pompe Disease

**DOI:** 10.1159/000528343

**Published:** 2023-01-09

**Authors:** Hacer Ilbilge Ertoy Karagol, Asli Inci, Sinem Polat Terece, Ayse Kilic, Fevzi Demir, Dilek Yapar, Gizem Koken, Ilyas Okur, Fatih Suheyl Ezgu, Leyla Tumer, Arzu Bakirtas

**Affiliations:** ^a^Department of Pediatric Allergy, Gazi University Faculty of Medicine, Ankara, Turkey; ^b^Department of Pediatric Metabolism and Nutrition, Gazi University Faculty of Medicine, Ankara, Turkey; ^c^Department of Biostatistics and Medical Informatics, Akdeniz University Faculty of Medicine, Antalya, Turkey

**Keywords:** Anaphylaxis, Pompe disease, Rapid drug desensitization, Recombinant alglucosidase alfa

## Abstract

**Background and Objective:**

Pompe disease (PD) is an inherited lysosomal storage disease that progresses with glycogen accumulation in many tissues, due to the deficiency of the acid-alpha glucosidase enzyme. Recombinant alglucosidase alfa (rhGAA) is the only disease-specific treatment option, in the form of enzyme replacement therapy (ERT). Anaphylaxis can develop with rhGAA. There is no study evaluating anaphylaxis and its management in PD in the long term. We aimed to evaluate the development of anaphylaxis and rapid drug desensitization (RDD) with rhGAA in children with PD.

**Materials and Methods:**

All children diagnosed and followed up in our institution with PD over 12 years between January 2009 and September 2021 were evaluated for development of anaphylaxis and RDD with rhGAA from medical records.

**Results:**

Fourteen patients, 64% of whom were female and diagnosed with PD (1 juvenile, 13 infantile types) during the study period included in the study. The median age at diagnosis was 3.2 months (1–40 months). The median follow-up time of the patients was 20 months (1–129 months). Thirteen patients were given rhGAA, one died before ERT. Four (30.8%) patients developed moderate to severe anaphylaxis, and RDD was applied with rhGAA. A total of 390 RDDs have been performed so far without any serious breakthrough reactions during all RDDs.

**Conclusions:**

Anaphylaxis with rhGAA is not rare and RDD with rhGAA is safe and effective in the long term.

## Introduction

Pompe disease (PD) is a rare inherited lysosomal storage disease that progresses with glycogen accumulation in many tissues, especially skeletal and cardiac muscle, due to the deficiency of the acid-alpha glucosidase (GAA) enzyme [1]. The infantile form, which first appears at the age of one, progresses rapidly and is fatal, while the later-onset forms progress more slowly and with serious morbidity [1]. There are no definitive data on survival in infantile-onset PD (IOPD) patients undergoing enzyme replacement therapy (ERT). However, it is well known that most untreated IOPD patients cannot survive beyond 1 year of age [2]. Mortality and morbidity have been reduced with the use of recombinant alglucosidase alfa (rhGAA), the only disease-specific treatment option, in the form of ERT [1]. Cross-reactive immune material (CRIM) determines the ERT response and ERT outcomes in IOPD. In CRIM-negative patients, the patients have no native enzyme production due to nonsense homozygous or compound heterozygous mutations that lead to an immune system reaction to exogenous GAA enzyme. In CRIM-positive patients, there is residual GAA enzyme activity. Response to ERT is better in CRIM-positive patients, and adverse reactions related to ERT are observed less frequently in this group [3].

Anaphylaxis can develop with this treatment, as with other drugs [4]. However, ERT-induced anaphylaxis in PD has been evaluated in a small number of case reports or in studies that include all patients with lysosomal storage disease [4, 5, 6, 7].

When anaphylaxis develops with ERT in these patients, rapid drug desensitization (RDD) is required since they definitely have to receive ERT. With this specific procedure, the drug causing anaphylaxis is started at very low doses and the target dose is reached by doubling the drug dose every 15–30 min. Thus, a temporary tolerance to the drug is established [8]. Similarly, data on desensitization with ERT are quite limited, as in the case of the development of anaphylaxis with ERT in PD patients [4, 5, 6, 7]. The aims of our study were to evaluate the frequency, clinical features, and management of anaphylaxis with rhGAA in children with PD and to evaluate the long-term follow-up of patients desensitized with rhGAA.

## Materials and Methods

### Patients

The medical records of the pediatric patients with PD who were being followed at the Pediatric Metabolism and Nutrition Clinic of our hospital between January 2009 and September 2021 were reviewed. Those who received rhGAA were evaluated for hypersensitivity reactions retrospectively. The demographic characteristics of the patients, the onset and timing of the reaction, diagnostic tests, management of the reaction, and the RDD protocol employed for the maintenance of ERT following the anaphylaxis were recorded. Also recorded were the follow-up times of the patients. Written informed consent was obtained from the patients' parents for RDD and skin testing with rhGAA.

### ERT Administration

The enzyme is administered at a dose of 20–40 mg/kg/dose weekly or biweekly, depending on the patient's clinical condition. The infusion is started at 1 mg/kg/h and gradually increased by 2 mg/kg/h every 30 min until a maximum rate of 7 mg/kg/h is reached. The premedication consisting of paracetamol (10 mg/kg), diphenhydramine (1 mg/kg), and methylprednisolone (1 mg/kg) is applied routinely 1 h before ERT.

### Definition and Phenotypes of Anaphylaxis

Anaphylaxis is an immediate type of hypersensitivity reaction. Symptoms occurring during enzyme infusion or within 1–6 h after the final enzyme administration are classified as immediate hypersensitivity reactions. The diagnosis of anaphylaxis was made according to the anaphylaxis 2020 practice parameter update, and its severity was defined according to the criteria of Brown SG [9, 10]. Phenotypes of anaphylaxis are classified according to symptoms and signs as type I IgE/non-IgE reactions (e.g., flushing, pruritus, urticaria, angioedema, throat tightness, shortness of breath, wheezing, nausea, vomiting, diarrhea, hypotension, desaturation, cardiovascular collapse), cytokine-release reactions (e.g., fever, chills/rigors, nausea, pain, headache, hypotension, desaturation), and mixed reactions [11].

### Diagnostic Allergy Work-Up

Skin prick tests (SPTs) were performed with 1/1,000, 1/100, 1/10, and undiluted forms of enzyme preparations, and intradermal tests (IDTs) were performed using 1/1,000, 1/100, and 1/10 dilutions of preparations. The enzyme preparations were initially tested on the volar forearm skin with the prick method. Results of the reactions were considered positive when the wheal diameter was more than 3 mm greater than that of the negative control, with surrounding erythema appearing within 20 min. When the SPTs revealed negative results, 0.02 mL of the enzyme preparations were injected intradermally on the volar forearm skin. Readings were made 20 min after the injections. Results were considered positive when there was a greater than 3 mm increase in the wheal diameter, accompanied by erythema. Positive control assays for SPTs and IDTs were conducted using histamine (10 mg/mL); 0.9% NaCl was used as a negative control sample for both SPTs and IDTs.

### Desensitization

A patient-specific RDD protocol was prepared for patients who developed anaphylaxis. According to the severity of the anaphylaxis, the starting dose in RDD was calculated to be between 1/1,000–1/1,000,000 of the total dose. The desensitization principles developed by Cernadas were applied [8]. All RDDs were performed by trained clinicians (allergy specialists) in the intensive care unit until the RDDs were successful.

### Breakthrough Reactions

Cutaneous symptoms (flashing, urticaria, angioedema), respiratory system findings (cough, wheezing, bronchospasm, stridor), hypotension, syncope, anaphylaxis, chills, rigors, fever, pain that occurred at any stage of RDD were defined as breakthrough reactions [8, 12].

### Statistical Analysis

Statistical analysis was performed using the SPSS 22.0 (Chicago, IL, USA) computer program. Categorical variables were given as numbers and percentages, and continuous variables were presented with medians (minimum, maximum) in the descriptive analysis. Two independent groups with and without anaphylaxis were formed. The χ^2^ test was performed to compare the categorical variables and the Mann-Whitney U test was used in the comparisons of non-normally distributed continuous variables.

## Results

### Demographics

Our cohort included 14 patients diagnosed with PD. The median age of diagnosis was 3.2 (1–40 months). One of the patients was in the juvenile type and the others were in the infantile type. Nine of the patients (64%) were girls. The median follow-up time of the patients was 20 months (1–129 months). One of the patients died before receiving ERT. Thirteen patients received ERT. Six of the 13 patients receiving ERT died due to PD-related reasons.

### Development of Anaphylaxis and RDD Application

Four (30.8%) of the 13 patients who could be treated with ERT developed anaphylaxis with rhGAA. A comparison of cases with PD, with and without an anaphylaxis reaction to ERT is given in Table 1. The clinical features of 4 patients who developed anaphylaxis are presented in Table 2. In case 3, anaphylaxis was moderate, while the others displayed severe anaphylaxis (Table 2). Only case 1 had a positive skin test with rhGAA; the others were negative. In case 2, anaphylaxis was the mixed type; the others were type I IgE/non-IgE anaphylaxis [11]. There was no history of other drug allergies among the patients and their families. Case 4 had asthma and her mother also had a history of asthma and venom anaphylaxis. Case 1 and case 2 have been previously reported [4, 13].

Desensitization with rhGAA was applied to these 4 patients. Currently, all the patients continue to receive ERT with a 4-dilution desensitization protocol and an initial dose ranging between 1/500,000 and 1/1,000,000 of the target dose. Detailed information for patients who underwent RDD is given in Table 2. Premedication was applied before RDD in case 2, while the others received no premedication. Methylprednisolone was administered as premedication, 13 h and 7 h before RDD, and methylprednisolone, diphenhydramine, and ranitidine were administered as premedication, 1 h before RDD. Premedication was gradually stopped in this patient.

In the follow-up, we successfully shortened desensitization time for case 3 by 3 h by increasing the infusion rate in the last step (increased from 4.2 mg/kg/h to 6.5 mg/kg/h). Thus, the desensitization time was reduced from 12.5 h to 9.4 h. The modified protocol applied to the patient is given in Table 3. We have not yet tried to modify the RDD protocols for the other 3 patients.

A total of 390 RDDs have been performed so far. The follow-up period of the patients who underwent RDD was between 12 and 129 months. Only in case 1, urticaria developed during the first two RDDs (breakthrough reaction rate: 2 reactions in 390 episodes of RDD: 0.5%). When the patients with and without anaphylaxis were compared in terms of age at diagnosis, gender, age at the onset of ERT, frequency of ERT administration, ERT dose, CRIM status, and parental consanguinity, no significant difference were found (*p* ≥ 0.05).

## Discussion

Our study is the first to evaluate anaphylaxis and its management with rhGAA in patients diagnosed with PD. Desensitization in PD patients was found to be highly effective and safe, and nearly 99% of RDDs were successfully performed for management of anaphylaxis with rhGAA.

The reactions that occur with rhGAA are usually reported under the title of adverse events, infusion-associated reactions, or hypersensitivity reactions and are not detailed in terms of anaphylaxis [7, 14, 15, 16]. In multicenter studies, the frequency of reactions with rhGAA ranges between 28.5 and 39.7% [14, 15, 16]. There are also references, however, that report the prevalence of severe allergic reactions with rhGAA in IOPD as low or absent. One of these is a research study that was conducted for a period of 52 weeks with 10 patients with IOPD who had received 20 mg/kg rhGAA biweekly; no severe adverse reactions were reported [17]. Another refers to an Italian expert panel review that states that approximately 1% of IOPD patients develop anaphylactic shock and/or cardiac arrest, while 5–14% of patients develop significant allergic reactions with rhGAA that involve at least two or three body systems [18]. Compared to these two reports, the rate of anaphylaxis in our study may seem higher. However, we think that the higher dose of rhGAA (40 mg/kg in 3 of the 4 patients compared to 20 mg/kg) and the shorter interval of the ERT application (weekly in 1 of 4 patients compared to biweekly) may have led to a higher rate of anaphylaxis. Unfortunately, we could not confirm this interpretation by statistical analysis due to the sample size used in the comparison of groups with and without anaphylaxis.

In our study, RDD with rhGAA was found to be quite safe and effective. No breakthrough reaction that we can describe as moderate to severe developed in any of the 390 RDD procedures we performed. The first case, which we initially reported in 2013, has been receiving ERT with RDD for more than 10 years. A cutaneous breakthrough reaction developed during RDD only in this case [4]. In the study of Turgay Yagmur et al. [7], RDD was applied to 3 out of 9 PD patients due to ERT-induced urticaria. It was reported that 2 of the patients developed moderate to severe anaphylaxis during RDD. Montelukast was added to the premedication administered before RDD in both patients, and no breakthrough reaction was observed. In this study, the reason a reaction developed during RDD could be the high initial dose of RDD. According to the given RDD protocol, the starting dose seems to be 1/80,000 of the total dose [7]. In our study, however, we kept the initial dose of RDD considerably lower than the total dose as recommended since the severity of anaphylaxis was moderate to severe [8].

Another important point to discuss is that RDD was performed without premedication in 3 of patients. Studies on RDD applications in pediatric patients are limited. In addition, RDD protocols in children are not standardized as in adults. Similarly, there is no consensus on premedication. It is not clear which drugs are to be used in premedication, when they will be administered (before or concurrently with RDD), and whether the drugs contribute to the success of RDD. One interesting case report in this context states that the authors used tranexamic acid in addition to other premedications at the time they desensitized their patients to rhGAA [19]. Unfortunately, it is not possible to comment whether the safe administration of rhGAA in this case report was due to desensitization or to the addition of tranexamic acid, or both. We started the desensitization with premedication as a precaution in the youngest patient but stopped the premedication gradually in subsequent desensitization episodes. In fact, premedication at the beginning of desensitization is not always recommended because it may mask early onset warning symptoms and signs of breakthrough reactions [8]. In addition, systemic corticosteroids as one of the premedications may negatively impact a patient's life when administered biweekly or weekly during ERT.

The mechanism of infusion-related reactions that occur during ERT in PD patients is not clear [13]. The key predictor for ERT tolerance is thought to be the CRIM status [3]. It is thought that CRIM status is highly correlated with treatment response, but immune response occurs in CRIM-negative infantile patients in a way that reduces treatment response [13]. From this point of view, only one of our patients who developed anaphylaxis was CRIM-negative. However, we could not detect any difference in disease characteristics (including CRIM status) when comparing those who developed anaphylaxis with those who did not. We believe that this may be related to the size of our sample. Most patients with IOPD develop antibodies to ERT depending on their CRIM status. There is, therefore, clearly a need for immune tolerance induction in CRIM-negative patients before ERT and during the treatment depending on the antibody development. CRIM-positive patients may also need immune tolerance induction during treatment in the case of the development of highly sustained antibody titers. Multiple immunomodulation protocols are being used and most of them involve rituximab, methotrexate, immunoglobulin, mycophenolate and cyclosporin A and/or azathioprine, and also plasmapheresis [20, 21, 22].

The strength of our study is that it is the first cohort study to present rhGAA desensitization in PD patients. There are already limited cohort studies on RDD in children. The limitations of the study were unavailability of serum tryptase and IL-6 measurements during anaphylaxis.

In conclusion, anaphylaxis with rhGAA is not rare in PD patients. Desensitization with rhGAA is safe and effective in the long term.

## Statement of Ethics

The Ethics Committee of the Gazi University approved the procedure (Code Number: 665/2021). The authors state that all the parents provided a written informed consent. The research was conducted ethically in accordance with the World Medical Association Declaration of Helsinki.

## Conflict of Interest Statement

The authors have no conflicts of interest to declare.

## Funding Sources

The study had no funding.

## Author Contributions

Hacer Ilbilge Ertoy Karagol, Arzu Bakirtas, Asli Inci, Sinem Polat Terece, Ayse Kilic, Fevzi Demir, Ilyas Okur, Gizem Koken, Fatih Suheyl Ezgu, and Leyla Tumer designed the study; Dilek Yapar analyzed the data, and Hacer Ilbilge Ertoy Karagol wrote the paper. All the authors discussed and approved the manuscript.

## Data Availability Statement

All data generated or analyzed during this study are included in this article. Further inquiries can be directed to the corresponding author.

## Figures and Tables

**Table 1:** Comparison of cases with PD with and without anaphylaxis to ERT

Characteristics	Patients with anaphylaxis (*n* = 4)	Patients without anaphylaxis (*n* = 9)	Total (*n* = 14)
Male, *n* (%)	1 (25)	4 (44.4)	5 (35.7)
Age at diagnosis, months	3 (1–40)	3 (1–15)	3.2 (1–40)
Positive CRIM status,^a^ *n* (%)	3 (75)	5 (71.4)	8 (72.7)
Parental consanguinity	4 (100)	7 (77.8)	12 (85.7)
Factors related with ERT^b^			
Age at the onset of ERT, months	4 (1–44)	4 (2–15)	4 (1–44)
High-dose ERT (40 mg/kg/dose), *n* (%)	3 (75)	2 (22.2)	5 (38.5)
Weekly ERT administration, *n* (%)	1 (25)	1 (11.1)	2 (15.4)

ERT, enzyme replacement therapy; CRIM, cross-reactive immune material; PD, Pompe disease.

a CRIM status was available in 11 patients.

b Calculated in 13 patients who received ERT.

**Table 2:** Characteristics of patients who developed anaphylaxis with rhGAA and underwent RDD

Case no.	Age at development of anaphylaxis, months	Present age, months	Initial reaction, signs, and symptoms	Type of anaphylaxis	Anaphylaxis developing dose	Administered dose and frequency when anaphylaxis develops	Infusion rate of developing anaphylaxis, mg/kg/min	RDD starting dose	RDD time, h	RDD performed, *n*
1	7	122	Urticaria, irritability, intractable cough, itching, stridor	Type I IgE mediated	11th	20 mg/kg Biweekly	0.016	1/1,000,000	11.7	264

2	5	34	Irritability, tachypnea, desaturation, cyanosis, fever, urticaria	Mixed type	8th	40 mg/kg Biweekly	0.116	1/500,000	19.6	56

3	48	72	Abdominal pain, recurrent vomiting, intractable cough	Type I IgE/non-IgE mediated	86th	40 mg/kg Biweekly	0.041	1/648,000	9.4	39

4	49	62	Urticaria, angioedema, tachypnea, cyanosis	Type I IgE/non-IgE mediated	10th	40 mg/kg Weekly	0.083	1/560,000	12	31

rhGAA, recombinant alglucosidase alfa; RDD, rapid drug desensitization.

**Table 3:** Intravenous desensitization protocol of Case 3

Solution type	Solution content			Concentration		
A	10 mL solution B + 90 mL 0.9% NaCl	0.002 mg/mL (1/1,000)
B	10 mL solution C + 90 mL 0.9% NaCl			0.02 mg/mL (1/100)		
C	10 mL solution D + 90 mL 0.9% NaCl			0.2 mg/mL (1/10)		
D	140 mL drug (700 mg) +210 mL 0.9% NaCl			2 mg/mL (1/1)		
**Step**	**Solution type**	**Rate, mL/h**	**Infusion duration, min**	**Volume infused per step, mL**	**Dose administered with this step, mg**	**Cumulative dose, mg**
1	A	2	15	0.5	0.001	0.001
2	A	4	15	1	0.002	0.003
3	A	8	15	2	0.004	0.007
4	A	16	15	4	0.008	0.015
5	B	4	15	1	0.02	0.035
6	B	8	15	2	0.04	0.075
7	B	16	15	4	0.08	0.155
8	B	32	15	8	0.16	0.315
9	C	8	15	2	0.4	0.715
10	C	16	15	4	0.8	1.515
11	C	32	15	8	1.6	3.115
12	C	64	15	16	3.2	6.315
13	D	16	15	4	4	14.31
14	D	32	15	8	16	30.31
15	D	52	15	13	26	56.31
16	D	52	341	296	592	648

Dose: 40 mg/kg/day; the patient's weight: 16.2 kg; total volume: 373.5 mL. NaCI, sodium chloride; alglucosidase alfa (Myozime^®^) = 50 mg = 10 mL.

## Notes

Edited by: Z. Zhao, Beijing.
